# Identifying Care Gaps and Referral Patterns in Acute Dermatology at Urgent Care Centers

**DOI:** 10.21203/rs.3.rs-4763112/v1

**Published:** 2024-08-14

**Authors:** Syed Minhaj Rahman, Acacia Bowden, Clayton B. Green, Alice P. Pentland, Christopher T. Richardson, Anna DeBenedetto, Molly Plovanich, Julie Ryan Wolf

## Abstract

Limited urgent access to board-certified dermatologists drives patients to seek dermatologic care at urgent care centers (UCC). UCC are staffed by clinicians with comparatively limited dermatology training, often resulting in lower quality care for acute dermatology conditions. This study investigates health care referral outcomes of patients seeking dermatologic care at UCC, examine the appropriateness of UCC dermatologic care, and assess the feasibility of referral management by eConsult. We utilize a retrospective cohort of 807 patients and a provider survey to examine referral outcomes of patients referred to University of Rochester Dermatology (UR-Derm), a tertiary care university-based teaching hospital, from UR UCC between January 1, 2021, and August 31, 2022. Outcomes for healthcare referrals included patient demographics, referral completion rates, UR-Derm appointment wait times, and diagnostic concordance rates between UCC and UR-Derm. Outcomes from the provider survey included appropriateness of UCC treatment plans, appropriateness of UCC referral to UR-Derm, and feasibility of referral management by eConsult. Patients who utilized UCC were predominately white (77.0%) females (53.9%) with a mean age of 37.9 years. Most patients referred by UCC did not complete an in-person UR-Derm evaluation (58.6%). Of those who did complete a UR-Derm visit, the average wait time was 38.3 days. Only 56% of UCC and UR-Derm diagnoses were concordant. Our surveyed dermatologists deemed 30% of the UCC treatment plans appropriate. The majority of referrals (83.5%) were viewed as manageable with an eConsult with only 10% of referrals requiring in-person visit. Several practice gaps exist in specialty care delivery in UCC and additional inefficiencies exist in the urgent referral process. These gaps could be addressed by targeted educational interventions and availability of dermatology consultation to support urgent care.

### INTRODUCTION

Each year, nearly 85 million Americans are treated for at least one skin condition.[1,2] Dermatologists are trained to diagnose and treat cutaneous disease, but their capacity to meet the urgent access needs of patients is limited, as evidenced by long appointment wait times. These access constraints redirect patients with acute or relapsing dermatological conditions to seek care from urgent care centers (UCC) and emergency departments (ED), shifting specialty care to clinicians with limited dermatology training. Studies have demonstrated knowledge gaps in dermatologic care delivered by non-dermatologists.[3,4] A 2017 study reported approximately 50% diagnostic concordance of dermatologic conditions between hospitalists and dermatologists.[3] Another study found that physicians selected at random from any other specialty had only a 25% chance of diagnosing an urgent skin condition with confidence.[4] Poor diagnostic concordance and diagnostic uncertainty can result in disease progression and inadequate symptom control. This may lead to increased patient health burden, unnecessary treatments, and ultimately increased healthcare costs.[5] Therefore, addressing the challenges with urgent access to dermatologic care is crucial for optimizing patient outcomes and enhancing the overall efficiency of our urgent healthcare delivery system. To assess UCC referral patterns and UCC dermatology related-practice patterns, we utilize a retrospective cohort and a board-certified dermatology provider survey to examine outcomes of UCC referrals to dermatology and the appropriateness of dermatologic care delivered by UCC clinicians.

## METHODS

### Study Design and Patient Population

A retrospective cohort and survey study was conducted under exemption by the University of Rochester (UR) Research Subjects Review Board (STUDY00007458). The UR Clinical and Translational Science Institute (CTSI) Informatics team extracted data from the electronic health record for all referrals to UR-Dermatology (UR-Derm) from eight UR-affiliated UCC locations between January 1, 2021 and August 31, 2022. All patients who had UCC visits for dermatologic conditions and a subsequent referral to UR-Derm were included. All UCC referrals evaluated in this study were referrals. There were no exclusion criteria based on gender, race, ethnicity, or age. Missing data from CTSI extraction were obtained from manual chart review. Data variables included: medical record number (MRN), date of birth (DOB), gender, race, ethnicity, residence zip code, marital status, insurance type, UCC referral site, UCC clinician type (e.g., MD/DO/MBBS, NP, PA), date of UCC visit, UCC visit diagnosis (i.e., ICD10 code and description), date of scheduled UR-Derm appointment, UR-Derm appointment completion status (i.e. “seen” or “not seen”), and UR-Derm visit diagnosis.

A retrospective study was utilized to assess outcomes of UCC referrals to UR-Derm including referral completion rates and wait times from UCC referral date to completed UR-Derm visit. Each referral was assigned a completion status of “seen” or “not seen”. Referrals were considered “seen” if the patient was evaluated at UR-Derm for the referred dermatologic condition. Referrals were considered “not seen” if a UR-Derm appointment was not scheduled, the patient refused or deferred an appointment, if the patient did not show up for their appointment, or if either the patient or the UR-Derm provider cancelled the scheduled UR-Derm appointment. Wait times from referral date to UR-Derm appointment date were categorized into “seen ≤ 14 days” or “seen > 14 days”.

Diagnostic concordance between UCC clinicians and UR-Derm clinicians was assessed in two ways. Diagnoses were considered concordant if the diagnosis made by UCC clinicians aligned with the diagnosis made by UR-Derm dermatologists in the subset of patients “seen”. Second, five board-certified dermatologists completed a REDCap survey to evaluate in detail the UCC practice patterns of 40 randomly selected UCC referrals to UR-Derm.

### REDCap Survey

A REDCap survey was administered to five board-certified dermatologists at UR-Derm. The survey presented 40 UCC referral cases (20 “seen” and 20 “not seen”) with de-identified UCC encounter details including history of present illness, UCC provider assessment note, UCC provider diagnosis, UCC provider plan, medications and orders placed during encounter, and clinical images of the skin condition provided for the referral. The reviewing dermatologists determined: 1) if the UCC provider’s treatment plan was appropriate; 2) if the UCC referral to UR-Derm was appropriate; and 3) if this patient could have been managed with a Dermatology eConsult. An eConsult is an asynchronous provider-to-provider electronic consultation in which the consultant (dermatologists) reviews the patient chart and makes care recommendations to be implemented by the referring provider. Treatment appropriateness and referral appropriateness were categorized by a 3-point scale into “not appropriate”, “somewhat appropriate”, and “appropriate”. Providers commented on their reasoning for treatments deemed "not appropriate" or "somewhat appropriate." For analysis of these comments, a manual search was conducted to identify trends, and justifications were reported if three or more dermatologists provided similar explanations for the case. EConsult management was categorized by a 3-point scale into “could be managed by eConsult”, “could be managed by eConsult with follow-up appointment with UR-Derm”, and “requires urgent in-person UR-Derm”.

### Data Handling and Statistical Analysis

For de-identification prior to analysis, patient DOBs were substituted with their respective age and each patient’s MRN was replaced with unique numeric Subject and Visit IDs. All statistical analyses were performed on deidentified data at a 5% level of significance using JMP16 Pro or Microsoft Excel. Descriptive statistics were used to characterize the patient population and mean wait times for UR-Derm visits. Primary analyses and secondary analyses involved Pearson chi-square tests and z-proportions tests to compare proportion differences in UCC referral completion rates in UR-Derm, diagnostic concordance, and REDCap survey results. We consolidated survey responses “appropriate” and “somewhat appropriate” into one “appropriate” category to perform additional analyses for treatment and referral appropriateness.

## RESULTS

### Patient Characteristics

Of the 807 UCC referrals to UR-Derm, most patients were predominantly Caucasian (77.0%) females (53.9%) with a mean age of 37.9 ± 20.8 years (Table 1). Only 6.7% of patients identified as Hispanic/Latino. Over 25% of patients with UCC referrals were diagnosed with "rash" (ICD 10 - R.21). The next most common UCC referral diagnoses included "abscess" (L02.91) (9.1%), “dermatitis” (L30.9) (7.0%), and "neoplasm of uncertain behavior" (D48.9) (6.9%). Interestingly, 48% of UCC referring clinicians were physician assistants, 31% were nurse practitioners, and 21% were MD/DO/MBBS.

Table 1
UCC Referrals to Dermatology

| Demographic Variable | Referrals N (%) |
|---|---|
| **Race** | |
| **Caucasian** | **621 (76.9)** |
| African American | 128 (15.9) |
| American Indian/ Alaskan Native/ Asian/ Other/ Unknown/ Refused | 58 (7.2%) |
| **Ethnicity** | |
| Hispanic/Latino | 54 (6.7) |
| **Not Hispanic/Latino** | **734 (90.8)** |
| Unknown/Declined | 19 (2.4) |
| **Sex** | |
| Male | 372 (46.1) |
| **Female** | **435 (53.9)** |

## Outcomes of UCC Referrals to Dermatology

From January 1, 2021 to August 31, 2022, there were 807 referrals from UCC to UR-Derm. Most UCC referrals (n = 473, 58.6%) were “not seen” by UR-Derm ($p < 0.0001$) (Table 2). Of the 334 referrals “seen” in UR-Derm, the majority (n = 210, 62.9%) were seen within 14 days ($p < 0.0001$) (Table 2). Although the median was 13 days, the average wait time of 38.3 days suggests a positive skew, which could be explained by limited short-term appointment availability and/or referrals that were triaged as non-urgent by UR-Derm and consequently scheduled for a later appointment. Diagnostic discordance between UCC and UR-Derm providers was observed in more than half of the UCC referrals “seen” in UR-Derm (56.0%, $p = 0.002$) (Table 2).

Table 2
UCC referral completion rates, wait times, and diagnostic concordance.

| Objective Variable | Referrals N (%) | p-value |
|---|---|---|
| **Completion Rates** | n = 807 | < 0.0001 |
| UCC Referrals Completed | 334 (41.3) | |
| **UCC Referrals Not Completed** | **473 (58.6)** | |
| **Wait Time** | n = 334 | < 0.0001 |
| **≤ 14 days** | **210 (62.9)** | |
| > 14 days | 124 (37.1) | |
| **Diagnostic Concordance** | n = 334 | 0.002 |
| UCC Diagnosis Match | 147 (44.0) | |
| **UCC Diagnosis No Match** | **187 (56.0)** | |

## Dermatologists Perspectives on UCC referrals

Our REDCap survey gathered the perspectives of five dermatologists on treatment appropriateness, referral appropriateness, and potential eConsult management appropriateness for 40 UCC referrals to UR-Derm (Table 3). Overall, most of the UCC treatment plans were considered at least “somewhat appropriate” by the dermatologist with only 24.5% considered inappropriate (Table 3). Of the cases with "inappropriate" or "somewhat appropriate" treatments, 35% of cases involved adjustments in steroid management, such as dosage, steroid class, or steroid quantity, by a majority of dermatologists (≥ 3 out of 5 dermatologists). Other common treatment adjustments included optimizing antibiotic usage or determining indications for bacterial or viral swabs. Rates of treatment appropriateness did not differ between UCC referrals that were “seen” or “not seen” in UR-Derm (74.5% vs. 75.5% respectively, $p = 0.352$), suggesting that knowledge gaps in therapeutic selection are independent of decision to refer to dermatology. The dermatologists believed that urgent referral was appropriate for only 26.5% of the UCC cases while 43.0% were “somewhat appropriate”, 23.0% were “not appropriate”, and 7.5% would require additional information to determine appropriateness. Potential justifications for "somewhat appropriate" and "not appropriate" referrals include cases that could be followed up with a patient's primary care physician, cases necessitating urgent care, or cases requiring a standard, non-urgent dermatology referral. Significantly more UCC referral cases that were “seen” in UR-Derm were deemed “appropriate” compared to those that were “not seen” in UR-Derm (83.7% vs 59.1%, respectively; $p = 0.0002$). Lastly, dermatologists reported that only 10.0% of the UCC urgent referral cases required an in-person evaluation by UR-Derm, while 83.5% of the referral cases could have been managed by the UCC clinician with UR-Derm eConsult ($p < 0.0001$) (Table 3).

Table 3
Survey results of 5 board-certified dermatologists depicting treatment, referral, and eConsult appropriateness for UC urgent referrals.

| Survey Results | Average % of cases |
|---|---|
| **Treatment Plan Appropriateness** | |
| Not Appropriate | 24.5% |
| Somewhat Appropriate | 43.5% |
| Appropriate | 30.0% |
| Additional Information Required | 2.0% |
| **Referral Appropriateness** | |
| Not Appropriate | 23.0% |
| Somewhat Appropriate | 43.0% |
| Appropriate | 26.5% |
| Additional Information Required | 7.5% |
| **EConsult Appropriateness** | |
| Requires Urgent Dermatology Visit | 10.0% |
| EConsult + Dermatology Follow-up | 45.5% |
| EConsult only | 38.5% |
| Additional Information Required | 6.5% |

## DISCUSSION

Our findings provide valuable insights into the current state of urgent dermatologic care and urgent referrals from UCC to dermatology. With 58% of UCC urgent dermatologic referrals not resulting in an in-person dermatology visit and a low 44% diagnostic concordance between UCC providers and dermatologists, we suspect these findings are negatively impacting patient outcomes.

Non-dermatologist clinicians are limited in their training regarding diagnosis and treatment of skin diseases, including urgent dermatologic conditions.[6] The limited dermatologic education in medical school and residency programs is well-documented.[2,9–11] Although published literature does not discuss the impact of limited dermatology training for UCC providers, there are several examples in the ED and primary care settings. Despite the fact that 3.3% of ED visits are for dermatologic conditions, a substantial 85% of internal medicine residents, who often work in ED, do not feel comfortable treating dermatological conditions.[7] Further, Dobry *et al*[8] found primary care practitioners' diagnoses were significantly discordant with teledermatologist diagnoses for non-urgent dermatologic cases.[8] Our study provides additional evidence of this gap in training by showing a low 44% diagnostic concordance between UCC clinicians and dermatologists in the subset of the cohort who completed the dermatology referral. Further, we found that 25% of UCC referral cases reviewed by dermatologists had inappropriate treatment plans for their dermatologic condition and 43% had only somewhat appropriate treatment plans. In our study, 79.1% of UCC referring providers were either physician assistants (PA) (48.2%) or nurse practitioners (NP) (30.9%). Since advanced practice providers require fewer clinical hours and less curriculum education than physicians, they may be less versed with dermatologic conditions, which in turn may lead to higher volumes of urgent referrals to Dermatology.[12,13]

Additional dermatology-specific training for UCC clinicians might help improve both diagnosis of cutaneous conditions with more appropriate treatment plans and urgent referral appropriateness. Early disease recognition and proper treatment play a vital role in optimal patient outcomes and referrals, specifically with urgent skin conditions.[4] Prior studies have shown that asynchronous teledermatology performed comparably to an in-person dermatologist for diagnosing and treating skin conditions of hospitalized patients.[3,14,15] Therefore, an eConsult service might also allow UCC clinicians to consult with a dermatologist to better diagnose and treat patients while simultaneously minimizing urgent referral burden. Dermatologists who participated in the survey provided qualitative feedback on the treatment plans from the UCC clinicians. Most responses regarding "somewhat appropriate" treatment plans indicated one of the following: the provider prescribed the correct medication, but the dosage was too low to achieve a therapeutic benefit; the correct medication was prescribed, but a longer prescription duration was required; or some laboratory tests were appropriately ordered, but additional laboratory tests were needed/too many laboratory tests were ordered. This feedback highlights the need for further education about the clinical management of dermatologic conditions, which could be provided by a dermatologist via eConsult. Additionally, the implementation of targeted educational interventions may enable UCC clinicians to make more accurate diagnoses and treatment plans for dermatologic conditions. Considering the existing evidence that supports improved patient outcomes through increased skin cancer detection via integration of a didactic dermatology curriculum, a similar approach of implementing a comprehensive curriculum for UCC providers to improve education regarding skin conditions may be effective.[16,17]

Our survey of five board-certified dermatologists assessed the potential of an eConsult model to improve the observed limited dermatology access. Most referred conditions were deemed not serious or complicated enough to warrant urgent referral for in-person consultation. We noted that 83.5% of UCC referrals could potentially be managed either by eConsult or by eConsult with dermatology follow-up. When considering the percentage of referrals that could be managed in UCC with dermatology consultation, this represents a significant opportunity to improve access to accurate dermatologic care. Implementation of an eConsult for UCC service would be a novel practice model that could also improve the high "not seen" rate of UCC referrals to dermatology by decreasing the number of in-person dermatology referrals for less serious skin concerns and increasing the proportion of truly urgent referrals “seen”.

In a previous study by Naka et al., 84% of store and forward dermatology eConsults from community health centers prevented the need for an in-person dermatology visit.[18] Numerous similar studies from multiple countries have reported rates ranging from 31%-77%, reinforcing the ability of eConsults to enhance access to dermatology via shortened wait times and decreased no-show rates, specifically in underserved populations.[5,18–23]. Our study reported that only 62.9% of UCC referrals were seen within 14 days, which may be due to lack of appointment availability. With only 26.5% of referrals deemed appropriate, there is a large volume of referrals from UCC that may be limiting access to care for patients with more severe skin disease. Decreasing the number of UCC referrals through the use of eConsults may increase appointment availability and ultimately increase access. Further, a notable 83.7% of UCC “seen” referrals were appropriate, compared to the 59.1% of referrals that were “not seen” (including no-show appointments and patient cancellations). A potential reason for this discrepancy could be that more appropriate UCC referrals were patients who felt their disease was severe enough to ensure being seen by dermatology. Regardless of being seen or not being seen by UR-Derm, sending urgent referral cases that are not truly urgent matters not only exacerbates administrative burden, but also exacerbates referral burden, further limiting dermatologic access for more appropriate referrals. However, to appropriately refer patients, providers may need to be better able to diagnose cutaneous conditions, or at least better determine urgency of the patient’s presentation. Our study findings on low diagnostic concordance between UCC clinicians and dermatologists support this notion as well as a recent study’s findings which indicate that non-dermatologists have a diminished ability to recognize urgent skin diseases contributing to misjudgment regarding appropriate urgent referrals.[4] Reducing the urgent referral stream may also be beneficial in planning urgent slots in dermatology clinics for patients with more severe disease. Potential implementation of a dermatology therapeutics educational curriculum focused on the treatment of acute cutaneous diseases may enhance training UCC providers and other non-dermatologist clinicians. Future studies should explore various process interventions, such as e-consultation, on-demand video visits, and/or urgent dermatology centers, to improve access in urgent settings.

## Limitations

As a single-site study, urgent referrals were extracted from UCCs that were only affiliated with the University of Rochester Medical Center network. Additionally, while we reviewed 100% of referrals from UCC over a 20-month period that were ultimately seen by UR-Derm (n = 807), we only included a small sampling of UCC referral cases (n = 40) in our more in-depth survey study to minimize respondent time and burden. Further, our survey respondents were all dermatologists from UR-Derm. These results may not reflect all urgent referral patterns from UCC to dermatology in other health systems but are informative for potential future larger studies across multiple health systems.

### Conclusions

Our study aligns with existing research supporting not only the lack of access to appropriate dermatologic care for urgent skin conditions but also the potential for managing a significant number of urgent referrals by implementing an eConsult system for UC centers. Our data highlights two concerning findings: 1) most urgent referrals fail to result in an in-person dermatology evaluation, and 2) there is poor diagnostic concordance between UCC clinicians and dermatologists. While these findings are consistent with previous studies regarding non-urgent cases[3,8,18,19,24,25] our study uniquely focuses on urgent referrals originating in UCC and further examines the practice patterns of urgent care clinicians. Due to the potential severity of urgent cases, long appointment wait-times and limited access to dermatology services increasingly restrict dermatology access for urgent referrals. This study highlights the knowledge gap in skin disease management in UCC, underscores the need for additional dermatologic training for UCC clinicians, and offers eConsults as a potential new care delivery model to increase access to urgent dermatologic care.

As a majority of UCC diagnoses and UR-Derm diagnoses were discordant and less than one third of urgent referrals were treated or referred appropriately, our study demonstrates that there is a need to improve diagnosis and treatment of dermatologic conditions by UCC clinicians. This could be managed by curating a targeted therapeutic-based dermatology management training for referring clinicians, building a stronger foundational knowledge regarding cutaneous conditions. Further, with only 10% of UCC referrals requiring urgent in-person evaluation by a dermatologist, implementing a dermatology eConsult system could not only increase access but also improve dermatologic care via improved diagnosis accuracy and treatment plans.

### Declarations

Funding: This article has no funding source.

Disclosures: The authors have no relevant conflicts of interest to declare. This article has not been published elsewhere, nor is it under consideration at any other journal. Abstract was accepted to the International Society of Investigative Dermatology Conference in Tokyo, Japan in May 2023.

**Acknowledgments:**

The authors would like to acknowledge the support of University of Rochester Clinical & Translational Science Institute for utilization of CTSI Informatics and REDCap (CTSA  UL1 TR002001)
